# PERSPECTIVE FROM A CARIBBEAN ISLAND, JAMAICA

**DOI:** 10.1093/geroni/igz038.1436

**Published:** 2019-11-08

**Authors:** Denise Eldemire-Shearer, Kayon M Donaldson-Davis, Julian McKoy Davis, Douladel M Willie-Tyndale

**Affiliations:** 1 Mona Ageing & Wellness Centre, Kingston, Jamaica; 2 Mona Ageing & Wellness Centre, Kingston 7, Saint Andrew, Jamaica; 3 Mona Ageing & Wellness Centre, Kingston 7, Kingston, Jamaica

## Abstract

Jamaica is prone to hurricanes and associated flooding. The older adult population is vulnerable given the increasing ageing population (12% over age 60), high rate of disability (20% - 30%) low literacy levels, increased feminization, and high numbers of old-old. This paper describes strategies employed in disaster management with a special focus on older persons as a vulnerable group. The significant contribution of social networks to disaster mitigation will also be discussed. Age-friendly strategies include older person representation on local disaster committees; mainstreaming the needs of older persons in community disaster plans; training of disaster workers on ageing and the special needs of older persons; maintaining community lists of frail, single and house-bound older persons. Social networks especially faith-based organisations are integral in disaster preparedness and recovery activities.

